# Radiation Dose and Fluoroscopy Time of Diagnostic Angiography in Patients with Spinal Dural Arteriovenous Fistula

**DOI:** 10.1007/s00062-021-01130-1

**Published:** 2022-01-07

**Authors:** Yigit Ozpeynirci, Christoph Trumm, Robert Stahl, David Fischer, Thomas Liebig, Robert Forbrig

**Affiliations:** Klinikum Großhadern, Institut für Neuroradiologie, Ludwig Maximilians University, Marchioninistr. 15, 81377 Munich, Germany

**Keywords:** Endovascular, Neurointervention, Dose reduction, Reference level, Diagnostic

## Abstract

**Purpose:**

Spinal dural arteriovenous fistulas (SDAVFs) represent the most common indication for a spinal angiography. The diagnostic reference level (DRL) for this specific endovascular procedure is still to be determined. This single-center study provides detailed dosimetrics of diagnostic spinal angiography performed in patients with SDAVFs.

**Methods:**

Retrospective analysis of all diagnostic spinal angiographies between December 2011 and January 2021. Only patients with an SDAVF who had baseline magnetic resonance angiography (MRA), diagnostic digital subtraction angiography (DSA), treatment and follow-up at this institution were included. Dose area product (DAP, Gy cm^2^) and fluoroscopy time were compared between preoperative and postoperative angiographies, according to SDAVF locations (common versus uncommon), MRA results at baseline (positive versus negative) and DSA protocols (low-dose, mixed-dose, normal-dose). The 75th percentile of the DAP distribution was used to define the local DRL.

**Results:**

A total of 62 spinal angiographies were performed in 25 patients with SDAVF. Preoperative angiographies (30/62, 48%) yielded a significantly higher DAP and longer fluoroscopy time when compared to postoperative angiographies (32/62, 53%) (*p* < 0.01). The local DRL was 329.41 Gy cm^2^ for a nonspecific (*n* = 62), 395.59 Gy cm^2^ for a preoperative and 138.6 Gy cm^2^ for a postoperative spinal angiography. Preoperative angiography of uncommonly located SDAVFs yielded a significantly longer fluoroscopy time (*p* = 0.02). The MRA-based fistula detection had no significant impact on dosimetrics (*p* > 0.05). A low-dose protocol yielded a 61% reduction of DAP.

**Conclusion:**

The results of the present study suggest novel DRLs for spinal angiography in patients with SDAVF. Dedicated low-dose protocols enable radiation dose optimization in these procedures.

## Introduction

Compared to cranial neuroendovascular procedures, spinal angiography is frequently associated with a greater amount of procedural radiation exposure, regardless of diagnostic or therapeutic nature, not only due to the different vascular anatomy but also the comparably larger volume and thickness of body parts being imaged [1].

Spinal dural arteriovenous fistulas (SDAVFs) are the most frequently encountered group of spinal vascular malformations (SVM), accounting for around 70% of all SVMs [2, 3] and are thus the most common indication for a spinal angiography. In this situation, selective spinal digital subtraction angiography (DSA) is essential and considered as the gold standard for a thorough understanding of the disease, establishing the therapeutic strategy and assessing the effectiveness of the treatment. Although a preceding magnetic resonance angiography (MRA) [4–6] is useful in locating the fistula level, a complete spinal angiography including catheterization of all feeding arteries from cranial to sacral levels, is occasionally needed. The significant number of injections may necessitate longer fluoroscopy times, a higher amount of radiation exposure and the use of additional contrast material. It is therefore essential to limit radiation exposure both to the patient and angiography team and to establish diagnostic reference levels (DRLs), as recommended by national and international advisory bodies [7, 8].

The DRL for spinal endovascular procedures is still to be determined by the National Federal Office of Radiation Protection, and reference values in the literature are scarce and variable [1, 4, 9–12]. We conducted a retrospective single-center study to analyze radiation dose, fluoroscopy time, and amount of contrast material in diagnostic spinal angiographies performed in a homogeneous group of patients presenting with SDAVFs in order to provide data valuable for the establishment of novel DRLs.

## Material and Methods

We retrospectively reviewed all diagnostic spinal angiographies performed at our institution between December 2011 and January 2021. Only patients with an SDAVF who had a preceding spinal MRA, diagnostic DSA, treatment and follow-up imaging at our institution were included. Exclusion criteria were the following: 1) spinal diagnostic angiograms performed as part of a therapeutic intervention, 2) angiograms combined with a complete 4‑vessel cerebral angiogram, 3) intracranial arteriovenous fistulas with perimedullary spinal venous drainage, 4) patients with a prior diagnostic DSA at another center who were referred only for treatment and 5) patients in whom the postoperative DSA was not carried out at our institution.

### Procedure

Prior to angiography, all patients with clinical and imaging findings suspicious of an SDAVF underwent an additional dedicated contrast-enhanced MRA (ceMRA) on a 3T MR scanner (Signa, GE Healthcare, Chicago, IL, USA) to detect the probable fistula level.

Angiographies were performed on a biplane angiographic unit (Axiom Artis Zee, Siemens Healthineers, Erlangen, Germany) by 5 consultant neuroradiologists with at least 6 yearsʼ experience in interventional neuroradiology. If the level of the fistula could be identified on MRA (MRA positive), selective catheterization started with the respective segment artery. Each preoperative angiography involved bilateral injections of at least five vertebral levels (contralateral segment artery at the fistula level and bilateral segment arteries from two adjacent segments above and below).

In MRA negative cases, catheterization began with any segment at the thoracolumbar junction and was continued until the feeder was identified. Segment arteries were scanned in standard anteroposterior projection at a rate of 2–4 frames per second (fr/s) and a field of view (FOV) between 11 and 21 cm. Once the fistula feeder was found, additional diagnostic runs were acquired at different angles and higher frame rates to display the fistula better, if necessary.

Postoperative angiography involved catheterization of the feeding segment artery and contralateral side at the same level as well as adjacent levels below and above on both sides. No rotational angiography was done.

Two DSA acquisition protocols preset by the manufacturer were used at the discretion of the neuroradiologist:

- Low dose (LD): 2 or 4 fr/s, 70 kV, pulse width 50 ms, dose 1.2 μGy/fr
- Normal dose (ND): 2 or 4 fr/s, 72.3 kV, pulse width 80 ms, dose 3 μGy/fr.

### Data Collection

Patient demographics, procedural data including procedure-related complications, and angiographic outcome at follow-up were retrospectively obtained from the medical charts. Imaging data and dose reports were retrieved from a dedicated picture archiving and communication system (PACS syngo.imaging, Siemens Healthineers). Procedural radiation exposure was measured as fluoroscopy time and dose area product (DAP, Gy cm^2^). The total DAP was determined by combining the DAP of fluoroscopy and DSA acquisitions. Fistula locations between the sixth thoracic and second lumbar vertebrae were regarded as common locations, as described by Krings et al. [2]. Fistulas located elsewhere represented the uncommon locations. For comparative group analysis, in cases with more than one preoperative angiography, procedural data from all preoperative angiographic studies were combined and considered as a single procedure. In cases with a residual fistula, only data from the final postoperative angiography confirming total cure were included.

### Statistical Analysis

Data were initially tested for normality using the Shapiro-Wilk test. Continuous variables were presented as means ± standard deviation (SD), percentages and ranges. Counts and percentages were used to represent categorical data. Mann-Whitney U tests were used to compare procedural data (DAP, fluoroscopy time and quantity of contrast material) between preoperative and postoperative angiographies. Dosimetric data of preoperative and postoperative angiographies between cases with fistulas at common and uncommon locations as well as between cases with feeders identified on preoperative ceMRA (MRA positive) and those not identified (MRA negative) were also compared with Mann-Whitney U tests. For both preoperative and postoperative angiographies, a Spearman correlation analysis was performed to determine the relationship between patient age and procedure DAP, fluoroscopy time and quantity of contrast material. The 75th percentile of the DAP distribution was used to define local DRLs for preoperative, postoperative and all spinal angiographies.

In addition, the impact of different DSA protocols on the DAP was examined. Three groups were formed for this purpose: 1) low dose (LD), 2) normal dose (ND) and 3) mixed dose (MD). One-way ANOVA analysis was used to compare the mean DAP, number of DSA acquisitions and dose index across these three groups (LD, ND and MD). The individual dose index was calculated for each patient using the following formula as previously described by Forbrig et al. [13]:

- Dose index (Gy cm^2^) = DAP of all DSA acquisitions/total number of DSA acquisitions

All calculations were performed using SPSS software version 25.0 (IBM, Armonk, NY, USA). A *p* value less than 0.05 was considered statistically significant.

## Results

Between December 2011 and January 2021 a total of 62 spinal angiographies, 30 (48%) preoperative and 32 (52%) postoperative, were performed in 25 patients with SDAVFs. Table 1 shows patient demographics and procedural data. In 5 (20%) patients, a repeat angiography was necessary to identify the fistula point due to excessive bowel gas (*n* = 1), technical challenge due to severe aortic atherosclerosis (*n* = 2) and termination due to discomfort of the patient (*n* = 2). In those patients, dosimetric data from two preoperative angiographies were summed up and represent the preoperative data.

**Table 1:** Patient demographics and procedural data

Patient data	
Mean age, years (range)	65 (45–82)
*Female sex*	6/25 (24%)
*Number of total DSAs*	62
Preoperative	30
Postoperative	32
*Surgical treatment*	22/25 (88%)
*Recurrence*	4/25 (16%)
*Fistulas at common locations (Th6-L2)*	17/25 (68%)
*Identification of the fistula point* *on the preangiographic ceMRA*	16/25 (64%)
*Complications*	1/62 (1.6%)

*DSA* digital subtraction angiography, *Th* thoracic, *L* lumbar, *ceMRA* contrast-enhanced magnetic resonance angiography

Of the patients 6 (24%) underwent more than 1 angiography postoperatively to confirm the fistula occlusion (residual or recurrent fistula, *n* = 4, persistent clinical and imaging findings despite fistula closure, *n* = 2). Only data from the final angiography showing fistula occlusion were considered in those cases.

One periprocedural complication occurred: in one patient a non-occlusive catheter-induced dissection of the fistula feeding segment artery occurred during the postoperative angiography.

**Fig. 1:**
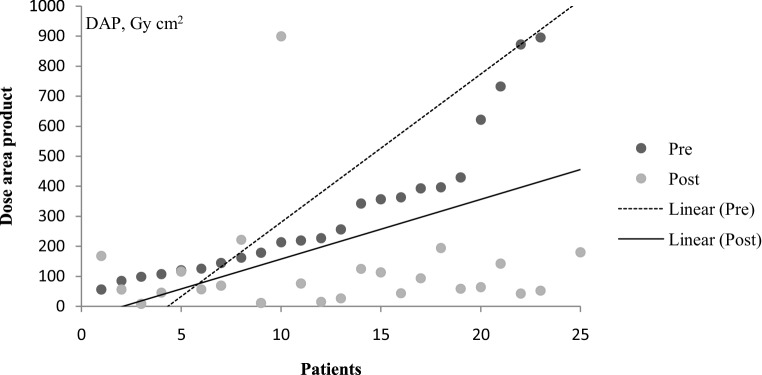
Scatter plot with linear regression lines reporting dose area product (DAP) values of preoperative (pre) (*dotted line*) and postoperative (post) (*solid line*) angiographies for each patient. The x-axis represents all patients from number 1 to number 25

**Fig. 2:**
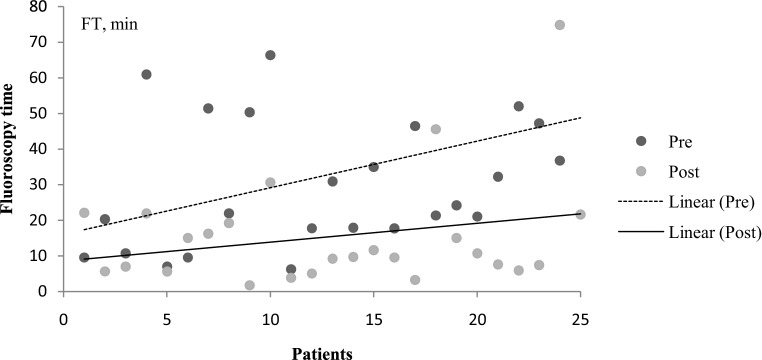
Scatter plot with linear regression lines reporting fluoroscopy times (FT) of preoperative (pre) (*dotted line*) and postoperative (post) (*solid line*) angiographies for each patient. The x-axis represents all patients from number 1 to number 25

Table 2 shows dosimetric data from preoperative and postoperative DSAs including fluoroscopy time, DAP, number of DSA runs, and contrast agent volume. Figs. 1 and 2 graphically represent DAP and fluoroscopy time of angiographies for each patient.

**Table 2:** Dosimetric data from spinal angiographies

	All angiographies (*n* = 62)	Preoperative angiographies (*n* = 30)	Postoperative angiographies (*n* = 32)
Mean DAP, Gy cm^2^ (range)	259.9 ± 360 (8.7–1724.2)	427.83 ± 428.02 (563.5–1724.2)	97.60 ± 106.17 (8.7–538.7)
Median DAP, Gy cm^2^	126	256.46	62.01
Mean FT, min (range)	19.6 ± 17.4 (1.8–111.5)	33.1 ± 23.5 (6.3–111.5)	11.1 ± 8.2 (1.8–38.8)
Median FT, min	16.9	24.2	7.6
Mean CV, ml (range)	142.9 ± 87.6 (40–500)	232 ± 123.2 (80–500)	96 ± 51.5 (40–270)
Median CV, ml	120	200	80
Mean number of DSA runs	16.6	26.3	7
Median number of DSA runs	10	20	6

*DAP* dose area product, *FT* fluoroscopy time, *CV* contrast material volume, *DSA* digital subtraction angiography

Preoperative angiographies had significantly higher DAP, fluoroscopy time, amount of DSA runs and volume of contrast material compared to postoperative angiographies (*p* < 0.01). In terms of total procedural DAP (38.5 vs. 51.8 Gy cm^2^, *p* = 0.3) or quantity of contrast agent (206 vs. 287 ml, *p* = 0.05), there was no significant difference between fistulas located at common and uncommon spinal levels in preoperative angiographies; however, fluoroscopy time (28.5 vs. 42.7 min, *p* = 0.02) and number of DSA acquisitions (24.8 vs. 29.3, *p* = 0.04) were significantly higher in the uncommon group. The DAP (411.1 vs. 457.5 Gy cm^2^, *p* = 0.4), fluoroscopy time (33.2 vs. 32.9 min, *p* = 0.7), number of DSA acquisitions (23.4 vs. 31.3, *p* = 0.3) and amount of contrast agent (215 vs. 262 ml, *p* = 0.4) were not significantly different between patients with and without a fistula point identified on preangiographic ceMRA.

In postoperative angiographies, there was no difference between fistulas located at common and uncommon spinal levels regarding the total procedural fluoroscopy time, DAP, amount of contrast agent or DSA acquisitions.

There was no significant correlation between age and DAP, fluoroscopy time, and the amount of contrast agent on preoperative and postoperative angiographies. The local DRL yielded 329.41 Gy cm^2^ for a nonspecific spinal angiography (*n* = 62), 395.59 Gy cm^2^ for a preoperative angiography (*n* = 30) and 138.6 Gy cm^2^ for a postoperative angiography (*n* = 32) (Table 3).

**Table 3:** Percentile values of dose area product (DAP) for preoperative and postoperative spinal angiographies. The 75th percentile defines the diagnostic reference level

Total DAP, Gy cm^2^	Preoperative (*n* = 30)	Postoperative (*n* = 32)	All (*n* = 62)
25th percentile	121.65	42.04	57.13
Median	223.14	66.52	126
75th percentile	**395.59**	**138.6**	**329.41**

An LD protocol was applied in 25/62 (40.3%) of the angiographies, an ND protocol in 27/62 (43.6%) and an MD protocol in 10/62 (16.1%) of the angiographies. The mean DSA acquisition count did not significantly differ between these protocol groups (LD: 13.6 vs. ND: 15.7 vs. MD: 14.6, *p* = 0.9). The mean dose index was significantly lower in the LD and MD groups than in the ND group (LD: 7.6 vs. MD: 10.2 vs. ND: 19.9, *p* < 0.01 and *p* < 0.05, respectively) and there was no difference between the LD and MD groups (*p* = 0.8). When compared to the ND protocol, the LD protocol resulted in a 61% reduction in DAP per single DSA run.

## Discussion

This study reports detailed dosimetric data from diagnostic angiographies performed in patients with spinal dural AVFs. The mean procedural DAP of 62 spinal angiographies performed in 25 patients was 260 Gy cm^2^, with a mean fluoroscopy time of 19.6 min and a mean volume of contrast material of 143 ml. As our local DRL, we propose 329.41 Gy cm^2^.

Spinal endovascular procedures, whether diagnostic or therapeutic, are frequently associated with a higher amount of radiation exposure compared to most intracranial fluoroscopically guided procedures. This is not only because of the difference in vascular anatomy but also the comparably larger volume and thickness of body parts being imaged [1]. Furthermore, complete spinal DSA, which is sometimes required to pinpoint the fistula location, entails selective catheterization of numerous segmental arteries in the neck, chest, abdomen and pelvis branching from various vessels. This may yield longer fluoroscopy times and the use of a larger volume of contrast material.

According to the literature [4–6] a high-resolution contrast-enhanced MRA of the spine may be helpful in locating the fistula site and consequently limit the angiographic effort. Luetmer et al. observed that MRA depicted a fistula in 20 of 22 patients presenting with an SDAVF. Only 14 cases had the level of the fistula included in the imaging volume, and in 13 (59%) cases the level and site of the fistula could be predicted within 1 vertebral level, resulting in a more than 50% decrease in fluoroscopy time and contrast agent volume [4].

Using preangiographic ceMRA, the level of the fistula was accurately detected in 16/25 (64%) of the patients in the present series; however, in preoperative angiographies no significant difference between MRA positive and MRA negative cases was found in terms of DAP, fluoroscopy time or contrast agent volume. This might be explained by the small population size, the angiographers’ various degrees of expertise and the unique technical challenges of each case.

SDAVFs may arise anywhere between the foramen magnum and sacrum. Krings et al. stated that in their experience more than 80% of all SDAVFs are located between the Th6 and L2 vertebral levels [2]. Likewise, in our study 17 fistulas (17/25, 68%) were detected between those levels.

Regarding preoperative angiographies, we found no significant difference between fistulas at common and uncommon levels in terms of procedure DAP and contrast agent volume; however, the mean fluoroscopy time was significantly higher in the group of fistulas found at uncommon levels (42.7 min versus 28.5 min), which is consistent when considering the angiographical routine workflow. If the preangiographic MRA is negative, as is the case with most of the fistulas at uncommon locations, we usually begin with selective catheterization of segmental vessels at the thoracolumbar junction and move on to sacral or upper thoracic segmental arteries if necessary. As a result, a longer fluoroscopy time is commonly required to locate the fistula in these cases; however, because the DAP of pulsed fluoroscopy is relatively low as compared to a DSA run, a longer fluoroscopy time does not necessarily imply a significantly increased radiation dose.

In the literature, radiation dose data from spinal diagnostic angiographies were commonly published from cohorts with heterogeneous pathologies [1, 4, 10–12, 14]. In contrast, we rather aimed to collect homogeneous data dedicated to a specific spinal vascular pathology. For example, Chen and Gailloud analyzed 302 consecutive spinal angiograms conducted in 288 patients over a 10-year period [11]. Only 25 patients (42%) in this collective had dural AVFs. The majority (95%) of angiograms were full spinal angiographic examinations. The average contrast amount was 110 ml and the fluoroscopy time 25 min. These values are comparable with those of our complete cohort (*n* = 62; contrast agent 143 ml, fluoroscopy time 19.6 min); however, Chen and Gailloud neither undertook any subgroup analysis nor presented data on radiation dose. Instead, they focused on procedure safety in terms of neurologic and systemic complications. In another study, Luetmer et al. reported a mean fluoroscopy time of 38 min and a mean volume of contrast agent of 219 ml in their series of preoperative spinal angiograms performed in 22 patients with SDAVFs [4]. These findings are consistent with our results from preoperative angiographies, with a mean fluoroscopy time of 33.1 min and a contrast agent volume of 232 ml.

Spinal vascular malformations can easily be overlooked on DSA, hence repeat angiography is not unusual. According to Gailloud, common factors are e.g. misinterpretation of segmental arteries, demonstration but not perception of lesions, unintentionally missing the level of the feeding vessel, deliberate limitation of the procedure (e.g., no pelvic injections) and poor or nonselective injections [14]. Moreover, excessive patient discomfort caused by prolonged examinations represents a common reason for the angiography being terminated early. If an initial study is negative, Gailloud et al. believe that the threshold for seeking a second view and/or repeat the angiography should be low [14]. Likewise, in five of our patients the fistula was eventually detected on the second preoperative angiography.

The ICRP (International Commission on Radiological Protection) and the European Directive 2013/59/EURATOM directives underline the need for justification of patient exposure to radiation and highlight the importance of both documenting the radiation dose of each examination and utilizing appropriate diagnostic reference levels [7, 8]. Institutions or individual practitioners gather radiation dose reports for a procedure performed in their own practice to use reference levels as a quality improvement tool. For the determination of local DRLs with acceptable 95% confidence intervals, Miller et al. proposed at least 30 studies of the same procedure [1].

The National Federal Office of Radiation Protection has not yet established a national DRL for spinal endovascular procedures [9]. Moreover, a dedicated literature query did not reveal officially established or recommended DRL for spinal angiographies in further European countries, the USA, South Korea or Australia [15–17].

Recently, Opitz et al. suggested a DRL for spinal angiographies performed in patients presenting with SDAVF. They presented dosimetric data for SDAVFs from 58 diagnostic spinal angiographies, revealing a DRL of 396.39 Gy cm^2^ [18], which is in line with our calculated values. Furthermore, two multicenter studies from France done by Etard et al. and Kien et al. reported DRLs for spinal angiographies [19, 20]. They included 123 and 171 spinal angiographies from various centers in France and proposed a DRL of 185 Gy cm^2^ and 483 Gy cm^2^, respectively; however, a pathology-based subgroup analysis was not carried out in their studies, disabling a reasonable comparison with our findings.

Our study has several limitations because of the monocentric design. Angiographies were performed using a single angiographic system from a single vendor (Siemens Healthineers). Furthermore, we only analyzed diagnostic spinal angiographies of SDAVFs, hence the provided dosimetric data cannot be generalized to other spinal endovascular procedures and pathologies; however, since dural AVFs are the most prevalent indication for a spinal angiogram, our data may be considered as guidance and cover the vast majority of patients undergoing this procedure. Finally, the dosimetric values provided in this study are not weight-corrected.

## Conclusion

In the present study we suggest novel DRLs for diagnostic spinal angiography in patients with dural AVF representing the most frequent spinal vascular pathology. Preoperative dosimetric values were significantly lower when compared to postoperative angiographic data. In preoperative angiography, the fluoroscopy time was significantly higher in patients with SDAVFs at uncommon locations, which in turn did not yield a significantly higher radiation dose. MRA-based fistula detection at baseline had no significant impact on the dosimetry values. A low-dose DSA protocol yielded a 61% reduction of radiation dose. We recommend further, ideally prospective multicenter studies in order to gather more detailed data of different angiography systems with novel dose reduction techniques as well as optimized DSA and fluoroscopy settings.
